# Influence of comorbidities not associated with fibromyalgia on neuropathic pain in patients with psoriatic arthritis: relationship with clinical parameters

**DOI:** 10.3389/fmed.2024.1331761

**Published:** 2024-01-24

**Authors:** Esther Toledano, Rubén Queiro, Luis Gómez-Lechón, Carolina Cristina Chacón, Cristina Hidalgo, Marta Ibañez, Agustín Díaz-Álvarez, Carlos Montilla

**Affiliations:** ^1^Department of Rheumatology, Hospital Clínico Universitario San Carlos, Madrid, Spain; ^2^Department Rheumatology, Hospital Universitario Central de Asturias, Oviedo, Spain; ^3^Department of Rheumatology, Francesc De Borja Hospital, Gandía, Spain; ^4^Department of Rheumatology, Hospital Universitario de Salamanca, Salamanca, Spain; ^5^Department of Anesthesiology, Hospital Universitario de Salamanca, Salamanca, Spain

**Keywords:** psoriatic arthritis, neuropathic pain, sleep quality, anxiety, depression, fatigue

## Abstract

**Objective:**

Neuropathic pain (NP) may influence disease activity assessment in patients with psoriatic arthritis, this relationship being traditionally based on the presence of concomitant fibromyalgia. We analyzed the influence of other comorbidities on NP and the relationship between pain and various clinical parameters.

**Methods:**

A cross-sectional study was conducted in patients diagnosed with psoriatic arthritis, excluding patients with a previous diagnosis of fibromyalgia, depression, anxiety, diabetes and/or dyslipidemia under treatment. NP was identified using the painDETECT questionnaire (score > 18). Obesity and related clinical parameters, anxious and depressive symptoms, sleep quality and fatigue were assessed as comorbidities. Disease activity was measured using the clinical Disease Activity Index for Psoriatic Arthritis (cDAPSA) in peripheral involvement, the ASDAS-PCR in axial involvement, functioning and disease impact were measured using the Health Assessment Questionnaire-Disability Index and 12-item Psoriatic Arthritis Impact of Disease questionnaire, respectively.

**Results:**

Overall, 246 patients were included (136 men; 55%). The mean age was 53.4 ± 11.0 years. Forty-two patients had NP (17.1%). Patients with NP had higher leptin levels (OR: 1.03, 95% CI: 1.007–1.056; *p* < 0.01) and poor sleep quality (OR: 1.20, 95% CI: 1.09–1.297; *p* < 0.001). Patients with NP also had greater fatigue NRS (6.2 ± 2.2 vs. 2.4 ± 0.19, *p* < 0.001). Patients with NP had higher cDAPSA score (17.3 ± 5.4 vs. 8.9 ± 6.5, *p* < 0.001), poorer functioning (1.1 ± 0.5 vs. 0.4 ± 0.5, *p* < 0.001) and greater disease impact (6.1 ± 1.7 vs. 2.6 ± 1.9, *p* < 0.001).

**Conclusion:**

NP was correlated with sleep quality and serum leptin and may be associated with worse disease activity, functioning and disease impact.

## 1 Introduction

Psoriatic arthritis (PsA) is a chronic disease associated with psoriasis, characterized by peripheral arthritis, axial manifestations, enthesitis and dactylitis. Advances in treatment have improved disease control and prognosis. However, some patients experience persistent pain despite low levels of inflammatory activity. This is an important issue as instruments used to measure disease activity and treatment response include subjective indicators related to perceived pain.

Pain in PsA is typically nociceptive due to activation of afferent nerve fibers in inflamed synovial tissue. Nonetheless, once this inflammation disappears, it is not uncommon that pain persists in the form of a complex neuropathic-like pain syndrome (1). Among other reasons, abnormal processing of pain in the central nervous system, also called central sensitivity, might be responsible for this type of pain (2). Central sensitivity may be explained by repetitive stimulation of nociceptors, thus increasing membrane excitability and leading to central nociceptive pathways disfunction (2, 3). This type of pain could be confused, due to its clinical characteristics of hyperalgesia and allodynia, with the typical pain of fibromyalgia, a common comorbidity in PsA, although it is included in different categories (neuropathic vs. nociplastic) (4, 5). On the other hand, in other diseases, such as rheumatoid arthritis (RA), it has been shown that it is not only patients with concomitant fibromyalgia who experience abnormal central processing of pain (6).

To date, few studies on PsA have explored the specific mechanisms of pain in this disease or its influence on the methods of assessing disease activity, and among them, the majority have indicated that the presence of comorbid fibromyalgia is the cause of such abnormal processing of pain (7–9). Indeed, in chronic inflammatory processes, there is not much evidence implicating other factors as a cause of neuropathic-pain (NP). On the other hand, relationships have been observed in other scenarios between NP and emotional and sleep disorders, or obesity-related processes (10, 11).

In this context, the aim of the study was to measure the frequency of PN in a group of patients with PsA, to determine its relationship with comorbidity and its influence on activity, functionality and impact of the disease.

## 2 Methods

### 2.1 Type of study

Cross-sectional study conducted at Salamanca University Hospital (Salamanca) and Francesc De Borja Hospital (Gandía, Valencia).

### 2.2 Population

#### 2.2.1 Inclusion criteria

We included consecutive patients over 18 years old with a diagnosis of PsA according to the ClASsification criteria for Psoriatic Arthritis (CASPAR) who were seen in outpatient rheumatology clinics between June 2019 and June 2020 and agreed to participate in the study (12).

#### 2.2.2 Exclusion criteria

We excluded patients who met the American College of Rheumatology (ACR) diagnostic criteria for fibromyalgia (2016) or had previously been diagnosed and treated for depression, anxiety, diabetes o dyslipidemia in order to avoid the possible influence on the measurements of emotional state and obesity (13, 14).

The study was approved by the ethics committee of Salamanca University Hospital (EO 17/19). Patients gave written informed consent before inclusion in the study and to publish the results derived from the research.

### 2.3 Variables assessed

#### 2.3.1 Demographic and clinical characteristics

Data were collected on the following variables: age, sex, time since diagnosis, smoking habits (smoker, former smoker, and non-smoker), treatment at the time of the study, patients that failed at least one bDMARDs due to inefficacy, form of the disease (peripheral, mixed, or axial), defining axial forms as inflammatory lower back pain with radiographic damage (sacroiliitis of at least grade 2 as per New York criteria and/or presence of syndesmophytes) (14), the presence of dactylitis (current or past), and the number of entheses involved as assessed using the Maastricht Ankylosing Spondylitis Enthesitis Score modified (mMASES). The original MASES focuses on 15 entheseal sites (the bilateral first and seventh costochondral joints, anterior and posterior superior iliac spines, iliac crests, and proximal insertion of the Achilles tendons, as well as the fifth lumbar spinous process) and this has been modified for PsA to include the plantar fascia, with scores ranging from 0 to 15 (15). It is easy to calculate because the final score is a straightforward sum of the enthesis involved (15).

#### 2.3.2 Type of pain

The NP was evaluated using the PainDETECT questionnaire (PDQ), a self-administered instrument developed to identify neuropathic pain. It includes seven 5-Likert scales (0 = never, 5 = very strongly) that investigate the qualitative characteristics of painful sensations (namely, burning, tingling or prickling, pain to light touch, sudden pain attacks, cold or heat sensitivity, numbness, and pain triggered by slight pressure) and an additional 2 points are awarded if the patient indicates radiating pain on a manikin. Lastly, there is a question that investigates the course of pain (scored from −1 to 1 depending on the pattern selected). The PDQ final score varies between −1 and 38. A score ≤ 12 indicates that a nociceptive component is likely and a score ≥ 19 indicates that a neuropathic component is likely, while the result is considered uncertain with intermediate scores (between 13 and 18) (16). For the statistical analysis, this last group was excluded, that is, we only compared patients reporting neuropathic pain with those reporting nociceptive pain.

#### 2.3.3 Variables related to obesity, emotional state, sleep quality, and fatigue

Obesity was measured using the body mass index (BMI). BMI is the result of dividing weight measured in kg by the square of height measured in meters. Obesity was considered if the BMI was greater than 30 (17). As a parameter associated with obesity, we measured leptin serum levels due to the association found with NP (18, 19). Leptin levels were measured by enzyme-linked immunosorbent assay (calibrated according to International Standard WHO/NIBSC 97/594, recombinant Leptin, using a Cobas e 411 analyzer with module E170 for modular analytics, and Cobas e 601 and e 602 analyzers).

We assessed the emotional factors using the Hospital Anxiety and Depression Scale (HADS). The HADS is a 14-item scale designed to identify people with anxiety and depression among individuals with medical conditions. Scores range from 0 to 21 for each subscale (HADS-D for depression and HADS-A for anxiety) and can be classified into one of three categories: normal (0–7), borderline abnormal indicating a possible clinical disorder (8–10), and abnormal indicating a probable clinical disorder (11–21) (20).

Sleep quality was analyzed using the Insomnia Severity Index (ISI). This is a self-administered questionnaire composed of seven items assessing the nature, severity and impact of insomnia. Responses are rated on a 5-point Likert-type scale ranging from 0 to 4, referring to the last month. The overall score ranges between 0 and 28 and can be interpreted with cut-off scores as follows: no clinically significant insomnia (0–7), subthreshold insomnia (8–14), moderate severity clinical insomnia (15–21) and severe clinical insomnia (22–28) (21).

Fatigue was measured using the second item of the 12-item Psoriatic Arthritis Impact of Disease questionnaire (PsAID-12) between 0 and 10 on a numerical rating scale (NRS) (22).

#### 2.3.4 Disease activity, functioning, and disease impact

In patients with peripheral involvement, disease activity was measured using the Clinical Disease Activity Index for Psoriatic Arthritis (cDAPSA) (23). This is a composite index for disease activity specific for PsA. As it is a clinical index, in this case CRP is not taken into account, unlike in DAPSA. It is calculated by summing the tender joint count (0–68), swollen joint count (0–66), the patient global assessment of disease activity score [between 0 and 10 on a numerical rating scale (NRS)], and pain NRS score (0–10). C-reactive protein level (CRP) (mg/dL) was also measured. In the case of patients with axial involvement, we used the Ankylosing Spondylitis Disease Activity Score with CRP (ASDAS-CRP) (24). Functional ability was measured using the Health Assessment Questionnaire—Disability Index (HAQ-DI) for peripheral involvement and the Bath Ankylosing Spondylitis Functional Index (BASFI) for axial involvement, and impact of the disease using the Psoriatic Arthritis Impact of Disease PsAID-12 (22, 25, 26).

### 2.4 Statistical analysis

Normally distributed variables were summarized using the mean and standard desviation (SD), and non-normally distributed variables by the median and interquartile range (IQR). Categorical variables as number and percentage. For continuous variables, comparisons between two groups were performed using Student’s *t*-test for independent samples in the case of normally distributed variables, and otherwise, the Mann–Whitney U test, while comparisons between several groups were carried out using one-factor analysis of variance for normally distributed data, and otherwise, the Kruskal-Wallis H test. Correlations between quantitative variables were assessed with Spearman’s correlation coefficient. *p* < 0.05 were considered statistically significant.

Demographic and clinical characteristics, comorbidities, activity, functional, and impact were compared in terms of the presence of NP.

A binary logistic regression (enter method) was performed, considering the presence of NP as the dependent variable and as independent variables the demographic and clinical characteristics (age, duration of the disease, biological treatment, mMASES) and the comorbidities (BMI, leptine, ISI, HADS-A, HADS-D) that could be related to NP (7, 8, 27, 28). The logistic regression analyses were adjusted for sex given evidence of sex-differences in NP and serum leptin level.

There were less than 3% missing values. A multiple imputation method was used.

The statistical analysis was performed using IBM SPSS version 20.

## 3 Results

### 3.1 Demographic and clinical characteristics

Among the 246 patients included, the mean age was 53 years old, and 136 were men (55%). The mean time since disease onset was 10 years. Nearly 70% of patients were being treated with conventional synthetic disease-modifying drugs (csDMARDs), while 1% (just three patients) were receiving a targeted synthetic disease-modifying drugs (tsDMARDs), 15% a biologic disease-modifying drugs (bDMARDs). Overall, 190 patients (77.2%) had peripheral involvement and 46 had mixed involvement (18.7%), the others having axial involvement. There were 42 cases of dactylitis, and the mean count of affected entheses was 1.BMI was correlated with leptin levels (r: 0.30; *p* < 0.001). A total of 164 patients (66.7%) reported nociceptive pain, 40 (16.3%) mixed pain and 42 (17.1%) NP. The baseline characteristics are summarized in Table 1.

**Table 1:** Baseline characteristics.

Patients	*n* = 246
Age (years) (m ± SD)	53.4 ± 11.0
Sex (man/woman)	136/110
Time since the onset of symptoms M (IQR)	9.7(10)
Smoking habit (smoker/former smoker/never smoker) (n)	64/109/73
Treatment with csDMARD (methotrexate/sulfasalazine/leflunomide) (n)	111/45/15
Treatment with tsDMARD (apremilast) (n)	3
Treatment with bDMARD (tumor necrosis factor inhibitor/secukinumab/ustekinumab) (n)	26/8/3
Patients that failed at least one bDMARDs due to inefficacy (n)	16
Clinical form (axial/mixed/peripheral) (n)	10/46/190
Dactylitis (yes/no)	42/204
mMASES M (IQR)	1(2)
PDQ (m ± SD)	9.8 ± 7.2
PDQ (nociceptive/mixed/neuropathic) (n)	164/40/42
BMI (m ± SD)	27.3 ± 4.9
Leptin (ηg/mL) M (IQR)	14.1(16.7)
HADS-A (m ± SD)	6.9 ± 4.1
HADS-D (m ± SD)	4.9 ± 3.9
ISI (m ± SD)	9.0 ± 5.9
Fatigue (m ± SD)	4.4 ± 2.6
cDAPSA (m ± SD)*	11.1 ± 7.0
SJC M (IQR)	1(2)
TJC M (IQR)	1(2)
Pain NRS (m ± SD)	3.5 ± 2.8
Activity NRS (m ± SD)	4.1 ± 2.6
CRP M (IQR)	0.2(0.6)
ASDAS-CRP (m ± SD)**	2.5 ± 0.9
HAQ M (IQR)*	0.5(0.8)
BASFI (m ± SD)**	3.2 ± 2.5
PsAID-12 (m ± SD)	3.6 ± 2.3

m, mean; n, number; SD, standard deviation; csDMARDs, conventional synthetic disease-modifying antirheumatic drug; tsDMARDs, targeted synthetic disease-modifying antirheumatic drug; bDMARD, biological disease-modifying antirheumatic drug; mMASES, modified Maastricht Ankylosing Spondylitis Enthesitis Score; PDQ, PainDETECT Questionnaire; HADS, Hospital Anxiety and Depression Scale; ISI, Insomnia Severity Index; cDAPSA, Clinical Disease Activity Index for PSoriatic Arthritis; SJC, swollen joint count; TJC, tender joint count; NRS, numerical rating scale; CRP, C-reactive protein; ASDAS-CRP, Ankylosing Spondylitis Disease Activity Score with CRP; HAQ, Health Assessment Questionnaire; BASFI, Bath Ankylosing Spondylitis Functional Index; PsAID-12: Psoriatic Arthritis Impact of Disease. *In patients with peripheral or mixed manifestations (*N* = 236). **In patients with axial or mixed manifestations (*N* = 56).

### 3.2 Relationship between demographic and clinical, obesity-associated variables, poor emotional state, poor quality sleep, fatigue, disease activity, functionality, and PsAID with NP

Female sex was related to NP (33.1% vs. 11.1%; *p* < 0.001), mMASES scores (2.5 vs. 0.8; *p* < 0.001) patients that failed at least one bDMARDs due to inefficacy (54.5% vs. 12.5%; *p* < 0.01). We found no difference with age (55.2 vs. 52.9; *p* = 0.23), disease evolution time (10.2 vs. 9.8; *p* = 0.81), smoking status- smokers, former smokers and never smokers- (22.2% vs. 22.5% vs. 15.9%; *p* = 0.56), treatment with csDMARDs-yes/no- (16.5% vs. 25.7%; *p* = 0.11) or bDMARDs-yes/no- (29.6% vs. 19.0%; *p* = 0.20), the clinical form-peripheral/mixed/axial- (23.9%vs. 7.9% vs. 11.1%; *p* = 0.06) or dactylitis-yes/no- (16.7% vs. 21.4%, *p* = 0.52).

There were no differences between NP and non-NP regarding BMI (28.2 ± 5.3 vs. 26.6 ± 4.9, *p* = 0.09). On the other hand, patients with NP had higher serum leptin levels and showed higher HADS-D, HADS-A and ISI scores than those with nociceptive pain. In the logistic regression analysis, leptin level (OR: 1.03, CI 95%: 1.007–1.056; *p* < 0.01) and ISI score (OR: 1.19, CI 95%:1.09–1.297; *p* < 0.001) were significant. Results are shown in Table 2.

**Table 2:** Results from univariate and multivariate analysis of the relationship between comorbidities and type of pain.

	Neuropathic	Nociceptive	Univariate	Multivariate
Age*	55.2 ± 8.6	52.9 ± 11.4	0.2	
Duration of the disease*	10.2 ± 7.8	9.8 ± 6.7	0.9	
Treatment with bDMARD (yes/no) (%)	29.6	19.0	0.2	
mMASES*	2.5 ± 2.7	0.8 ± 1.4	0.001	
BMI*	28.2 ± 5.3	26.6 ± 4.9	0.09	
Leptin (ηg/mL)*	26.8 ± 21.6	14.2 ± 13.5	0.001	OR: 1.03(1.007–1.056); *p* < 0.01**
HADS-D*	7.6 ± 4.10	3.9 ± 3.4	0.001	
HADS-A*	9.8 ± 3.7	5.8 ± 3.9	0.001	
ISI*	14.5 ± 6.1	7.3 ± 4.9	0.001	OR: 1.19(1.09–1.297); *p* < 0.001**

HADS-D and HADS-A, Depression and Anxiety subscales of the Hospital Anxiety and Depression Scale respectively; ISI, Insomnia Severity Index. *Values in cells represent (mean ± SD). **Nagelkerke R^2^: 0:52.

Patients with NP had greater levels of fatigue than patients with nociceptive pain (6.2 ± 2.2 vs. 2.4 ± 0.19; *p* < 0.001).

Patients with NP had higher cDAPSA scores than patients with nociceptive pain (17.3 ± 5.4 vs. 8.9 ± 6.5; p < 0.001). The components of cDAPSA are listed by type of pain in Table 3. No differences were found with CRP (mg/dl) (0.8 ± 1.0 vs. 0.6 ± 0.9; *p* = 0.8).

**Table 3:** Components of the clinical disease activity index for psoriatic arthritis and C-reactive protein level by type of pain.

	Neuropathic pain	Nociceptive pain	*p*
TJC	2.7 ± 2.4	1.4 ± 1.9	0.001
SJC	1.2 ± 0.9	1.1 ± 1.2	0.7
Activity NRS	6.2 ± 2.2	3.2 ± 2.4	0.001
Pain NRS	6.2 ± 2.2	2.5 ± 2.3	0.001

TJC, tender joint count; SJC, swollen joint count; NRS, numerical rating scale. Values in cells represent (mean ± SD).

Among patients with axial manifestations, those with NP had higher ASDAS-CRP scores (3.6 ± 0.4 vs. 2.2 ± 0.9; *p* < 0.005). Lastly, patients with NP had poorer functioning both in patients with peripheral (1.1 ± 0.5 vs. 0.4 ± 0.5; *p* < 0.001) and axial involvement (4.4 ± 2.2 vs. 2.1 ± 2,0; *p* < 0.001) and higher PsAID-12 scores (6.1 ± 1.7 vs. 2.6 ± 1.9; *p* < 0.001) than those with nociceptive pain.

## 4 Discussion

In this study we have confirmed the hypothesis that NP may be associated by other comorbidities such as obesity and associated factors, anxiety, depression, sleep disorder and fatigue and that this type of pain may be associated with disease activity, functioning and disease impact. Furthermore, by excluding patients with concomitant fibromyalgia we can suggest that NP is inherent to PsA.

In particular, we found that sleep quality and serum leptin level explained 50% of the variation in NP in patients with PsA. To our knowledge, no other data have been reported relating sleep disorders to the presence of NP in patients with PsA. These results can be understood from a pathophysiological perspective, as it has been demonstrated that sleep deprivation has a hyperalgesia effect, secondary to impairments in descending pain modulatory systems, that affects nociceptive responses (10, 29). Further, improvements in sleep quality have been linked to reductions in chronic musculoskeletal pain over the long term (30).

In our study, leptin level was correlated with NP. In line with this, obesity has been reported to influence chronic pain (11). In a recent study, Ballegaard et al. indicated that obesity is a predictor of poor prognosis in terms of response to treatment, these results having been obtained considering the influence of obesity on pain rather than on inflammation (27). In a later study, BMI was a factor related to PsA patients persisting with symptoms despite having failed two b/ts DMARDs. In our work we found that patients who had failed more than one bDMARD had a higher frequency of NP. However, they did not have a higher BMI (28.4 ± 5.2 vs. 26.9 ± 4.7, *p* = 0.10, data not shown). The different classification of “non-responders” could be responsible for these results (31).

Although not specifically concerning NP, a recent study in PsA patients related serum leptin level with pain measured by VAS score and tender joint count (28). Experimental research suggests that leptin interacts with neuronal transmission by promoting the hyperexcitability of key neuronal pathways involved in the onset of NP (18). Higher levels of serum leptin have been reported in patients with NP following spinal cord injury (19). These studies give strength to our finding regarding the leptin-NP relationship in PsA.

A relationship between NP and fatigue was observed previously in the DANBIO registry, where patients with fatigue showed higher PDQ scores (9.0 [6.0–14.0] vs. 17.0 [13.0–23.0], *p* < 0.001), findings that might indicate that the source of the NP was related to central hypersensitivity (32). In our study, unlike the DANBIO registry, we excluded patients with fibromyalgia suggesting that such a process of central hypersensitivity seems to be characteristic of PsA.

In our patients, NP was associated with disease activity, functioning and disease impact. Although these results are compatible with those of most studies, we found that the percentage of patients with neuropathic pain (17.1%) was lower than in previous studies, which have reported rates of over 25% (7, 9). These differences may be explained by our exclusion of patients with fibromyalgia. In the DANBIO registry, patients with NP had higher peripheral disease activity as measured using the Disease Activity Score in 28 Joints with CRP level (3.7 vs. 2.4, *p* < 0.001). Like our study, the authors did not find any relationship between NP and age or time since diagnosis (7). In patients with RA, it has been proposed that hypersensitivity to pain may be related to disease duration, a relationship not observed in our study (33).

Consistent with our results, Ramjeeawon and Choy found NP to be related to the patient’s global assessment, pain intensity, tender joint count and total joint count. These authors also found an association with anxiety and depression (8). However, an Italian study did not find any correlations between neuropathic pain and cDAPSA, HAQ or PsAID. Excluding patients with fibromyalgia, although the rate of NP was similar to that in our study (13.63%), the authors only found an association with HAQ-DI (*p* < 0.04) (9). This study could be underpowered due to small sample size.

One of the limitations of our study is related to its design. Given its cross-sectional nature, we are unable to establish causality. Indeed, we understand that some of the relationships observed may be bidirectional; that is, the presence of neuropathic pain may trigger sleep disorders, and vice versa. It is noteworthy that patients undergoing knee replacement surgery, neuropathic pain predicted the incidence of sleep disorders (34). Nonetheless, in that case, it remained unclear whether the NP was attributable to damage to sensitive terminal nerve fibers from the intervention or to repeated stimulation of nociceptors due to the symptoms of arthritis. The previous reasoning may also be applied to the relationship between leptin levels and NP.

Other limitation is that we did not select our study population randomly. On the other hand, the inclusion of consecutive patients over 1 year means the sample should be reasonably representative, given that patients are seen routinely at 3- to 6-month intervals, and hence, 1 year was sufficient time for them to be assessed at least once during the recruitment period.

As strengths, it is a study with a large number of patients, recruited from two centers. As mentioned above, although there are some studies similar to ours, the authors did not exclude patients with concomitant fibromyalgia, so it cannot be assumed that NP is inherent to PsA. Finally, to our knowledge, there is no study that has analyzed the influence of NP according to the activity of the type of manifestation (peripheral or axial).

## 5 Conclusion

In summary, NP may be related to comorbidities such as poor sleep quality and obesity-associated factors, and as patients with fibromyalgia were excluded, this specific type of pain seems an additional characteristic of PsA. However, studies of higher methodological quality are needed to substantiate this claim. Patient-reported outcomes including their assessment of disease activity were associated with NP. This finding may be important for avoiding overtreatment with biologics and guiding the prescription of treatments targeting potential causes of pain (for example, drugs to improve sleep quality and/or specific for NP).

## Data availability statement

The original contributions presented in the study are included in the article/supplementary material, further inquiries can be directed to the corresponding author.

## Ethics statement

The study was approved by the Ethics Committee of the Hospital Universitario de Salamanca (EO 17/19). The studies were conducted in accordance with the local legislation and institutional requirements. The participants provided their written informed consent to participate in this study.

## Author contributions

ET: Writing – original draft, Data curation, Investigation. RQ: Writing – review & editing, Conceptualization. LG-L: Writing – review & editing, Conceptualization, Data curation, Investigation, Writing – original draft. CC: Writing – review & editing, Writing – original draft. CH: Writing – review & editing, Writing – original draft. MI: Writing – review & editing, Writing – original draft. AD-Á: Writing – review & editing, Writing – original draft. CM: Funding acquisition, Investigation, Writing – original draft, Writing – review & editing, Conceptualization, Data curation, Supervision.
